# Brain-penetrant complement inhibition mitigates neurodegeneration in an Alzheimer’s disease mouse model

**DOI:** 10.1093/brain/awae278

**Published:** 2024-08-31

**Authors:** Wioleta M Zelek, Ryan J Bevan, Jacqui Nimmo, Maarten Dewilde, Bart De Strooper, Bryan Paul Morgan

**Affiliations:** School of Medicine, UK Dementia Research Institute Cardiff, Cardiff University, Cardiff CF14 4XN, UK; School of Medicine, UK Dementia Research Institute Cardiff, Cardiff University, Cardiff CF14 4XN, UK; School of Medicine, UK Dementia Research Institute Cardiff, Cardiff University, Cardiff CF14 4XN, UK; Therapeutic and Diagnostic Antibodies, Pharmaceutical and Pharmacological Sciences, KU Leuven, Leuven 3000, Belgium; PharmAbs, The KU Leuven Antibody Center, KU Leuven, Leuven 3000, Belgium; Centre for Brain and Disease Research, KU Leuven and VIB Leuven, Leuven 3000, Belgium; UK Dementia Research Institute, University College London, London WC1E 6BT, UK; School of Medicine, UK Dementia Research Institute Cardiff, Cardiff University, Cardiff CF14 4XN, UK

**Keywords:** neuroinflammation, therapy, drug delivery, blood–brain barrier, mouse model

## Abstract

Complement activation is implicated in driving brain inflammation, self-cell damage and progression of injury in Alzheimer’s disease and other neurodegenerative diseases. Here, we investigate the impact of brain delivery of a complement-blocking antibody on neurodegeneration in an Alzheimer’s mouse model. We engineered a brain-penetrant recombinant antibody targeting the pro-inflammatory membrane attack complex.

Systemic administration of this antibody in APP^NL-G-F^ mice reduced brain levels of complement activation products, demonstrating successful brain entry and target engagement. Prolonged treatment decreased synapse loss, amyloid burden and brain inflammatory cytokine levels, concomitant with cognitive improvement compared to controls. These results underscore the potential of brain-penetrant complement-inhibiting drugs as promising therapeutics, targeting downstream of amyloid plaques in Alzheimer’s disease.

## Teaser

Zelek *et al.* develop a brain-penetrant recombinant antibody that targets the pro-inflammatory membrane attack complex. In an Alzheimer’s disease mouse model, systemic administration of this antibody lowered brain levels of complement activation products, and reduced neurodegeneration, synapse loss, and amyloid burden.

## Introduction

Alzheimer’s disease (AD) is the most prevalent neurodegenerative disease (NDD) and commonest cause of dementia. To date, only a handful of drugs have been approved for the treatment of AD; most provide only symptomatic relief and have no impact on the disease course.^1-5^ In the quest for disease-modifying drugs, most attention has focused on removing amyloid, culminating in the recent US Food and Drug Administration (FDA) approval of the anti-amyloid-β (Aβ) antibodies aducanumab, lecanemab and donanemab, despite their limited impact on rate of disease progression and toxicity issues.^6-9^ There remains an overwhelming need for new drugs that slow or stop progression in AD, and this requires the identification of new targets. Numerous recent reports, including genetic, biomarker and animal model studies, have implicated neuroinflammation as a driver of pathology in AD^10-12^; therefore, targeting neuroinflammation early in the disease process is an attractive strategy.

Complement is a key component of immune defence and a critical driver of inflammation in health and disease; indeed, modulating complement activation has proven effective in treating diverse inflammatory diseases.^13^ Complement comprises three main pathways: the classical pathway, activated by immobilized antibody; the lectin pathway, activated by specific sugars; and the alternative pathway, activated spontaneously or by the other two pathways to amplify activation. All activation pathways converge on C3 cleavage, which initiates terminal pathway activation. Inflammatory products (C3a, C5a) and opsonins (C4b, C3b) are generated from the activation pathways, while the terminal pathway yields the cytolytic membrane attack complex (MAC). Several lines of evidence implicate complement in AD pathogenesis. The genes encoding the complement system proteins complement receptor 1 (CR1) and clusterin are top hits in AD genome-wide association studies (GWAS),^14,15^ while fluid (plasma and CSF) and tissue complement biomarker studies in AD provide evidence of complement dysregulation early in the disease course.^10,16,17^ Complement deficiency in animal models of AD has been shown to prevent or ameliorate disease.^18-21^ Collectively, the data implicate complement as a driver of neuroinflammation and resultant neurodegeneration in AD.

Complement offers many opportunities for drug inhibition^18,19,22^; however, complement provides critical defence against bacterial infections and is crucial to immune complex handling and immune defence, with roles including priming innate and adaptive immunity, regulating metabolism and tailoring neural development.^23-25^ Potential impact on these protective and homeostatic roles influences choice in selecting drug targets, particularly for long-term therapy. Targeting in the terminal pathway, responsible for MAC formation, spares most protective and homeostatic functions, including opsonic and chemotactic activities. Indeed, a drug targeting MAC formation at the stage of C5, eculizumab, has been in the clinic for nearly 20 years.^13,26^ Several other complement blockers have recently been FDA approved. The majority of these are antibodies (∼150 kDa) or other large molecules, unable to penetrate the blood–brain barrier (BBB) efficiently because of their size; a few are small molecules but are not designed to be BBB permeable.^13,18^ For effective inhibition of complement in the brain, we need new complement-blocking drugs that can penetrate the BBB.

We have developed a toolbox of novel monoclonal antibodies (mAbs) that block MAC formation by targeting C7; these are efficient inhibitors of human and rodent complement *in vitro* and *in vivo.*^27^ Here, we describe the generation and characterization of a recombinant version of one of these mAbs, clone 73D1, chosen for strong inhibition of mouse C7 and modified for efficient BBB penetrance. The recombinant mAb was expressed fused to a nanobody (Nb62) against the transferrin receptor (TfR); Nb62 has previously been shown to facilitate brain entry of cargo peptides and proteins.^28,29^ The Nb62-linked recombinant mAb (Nb62-r-mAb) retained full complement blocking activity and was BBB penetrant *in vivo* in mice. When administered systemically to AD model (APP^NL-G-F^) mice, Nb62-r-mAb reduced complement activation in brain, protected from synapse loss, reduced brain inflammation and amyloid load and improved cognition when compared to treatment with a BBB non-penetrant control comprising the same r-mAb linked to an irrelevant nanobody (control-r-mAb). The data suggest that MAC blockade in brain might be an effective therapy for AD.

## Materials and methods

### Reagents and sera

All chemicals, except where otherwise stated, were obtained from Fisher Scientific or Sigma Aldrich and were of analytical grade. All tissue culture reagents and plastics were from Invitrogen Life Technologies. Sheep erythrocytes in Alsever’s solution were from TCS Biosciences. Human and animal sera were prepared in-house from freshly collected blood. For human serum, blood was clotted at room temperature (RT) for 1 h, then placed on ice overnight for clot retraction before centrifugation and harvesting of serum. For mouse serum, blood was placed on ice immediately after harvest and clotted for 2 h on ice before serum harvest. Sera were stored in aliquots at −80°C and not subjected to freeze–thaw cycles.

### Animals

Animals were group-housed in open topped cages with 12-h light-dark cycles and food and water available *ad libitum*. Heterozygous C7 deficient mice (C57BL/6NJ-C7^em1(IMPC)^ J/Mmjax C7+/−; Jackson ImmunoResearch) were bred in-house to obtain homozygous C7-deficient mice.^27^ APP^NL-G-F^ knock-in mice carrying the APP Swedish (KM670/671NL), Iberian (I716F) and Arctic (E693G) mutations were kindly donated by Dr Takaomi Saido under a materials transfer agreement.^30^ These mice develop aggressive amyloidosis with plaques, surrounded by activated microglia and astrocytes, present from ∼2 months; synapse loss is present from 4 months and cognitive impairment apparent from 6 months (https://www.alzforum.org/research-models/app-nl-g-f-knock). For all the experiments, a single sex (males) was used, because the well-described differences in complement activity between male and female mice adds a confounder in mixed sex studies.^31,32^ All animal procedures were performed in accordance with UK Home Office Animals Scientific Procedures Act 1986 and local institutional guidelines. At appropriate time points, mice were humanely sacrificed with increasing CO_2_ concentration and death confirmed by permanent cessation of circulation. Whole blood was collected by transcardial puncture and processed as described above. Mice were perfused intracardially with PBS, brains removed and cut sagittally, one half fixed in paraformaldehyde (PFA; 1.5%) for immunocytochemistry and DiOlistic spine labelling and the other half snap frozen for protein analyses.

### Generation of recombinant monoclonal antibodies

**Figure 1:**
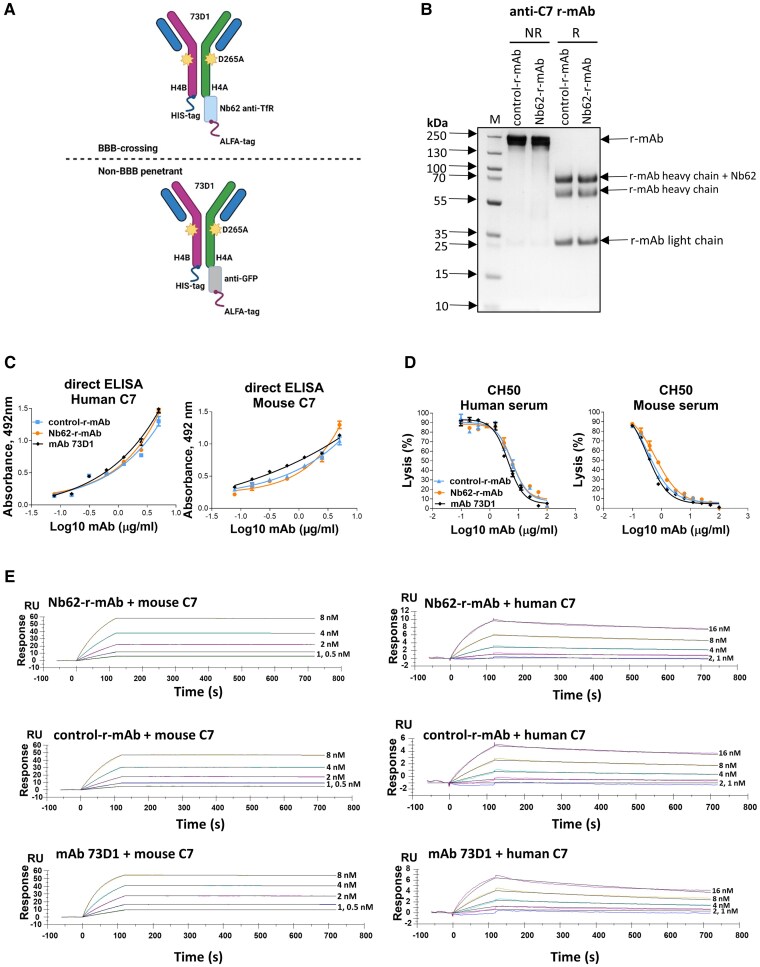
**Generation and characterization of the brain penetrant anti-C7 recombinant monoclonal antibodies (r-mAbs).** (**A**) Cartoon representing the design of the Nb62-r-mAb (*top*) and control-r-mAb (*bottom*). Complementary determining regions (CDRs) from mAb 73D1 were grafted onto a mouse IgG2a framework modified (D265A) to ablate Fc receptor binding. Nanobodies, Nb62 against low-affinity transferrin receptor (TfR) for the test, anti-GFP for the control, were expressed at the carboxy terminus of one heavy chain. ALFA-tag and 6-His tag were included in each construct for use in detection and purification. (**B**) Sodium dodecyl sulfate-polyacrylamide gel electrophoresis (SDS-PAGE) of Nb62-r-mAb and control-r-mAb. Proteins were purified on protein G then run on 7.5% acrylamide gels, either non-reduced (NR) or reduced with 5% β-mercaptoethanol (R). S**e**parated proteins were stained with Coomassie Blue. NR = ∼150 kDa MW intact r-mAb; R = 55 kDa MW r-mAb heavy chain, 25 kDa MW r-mAb light chain and ∼70 kDa MW r-mAb heavy chain plus nanobody. (**C**) Direct ELISA. Human and mouse C7 were immobilized on wells then native mAb, Nb62-r-mAb or control-r-mAb added in a dilution series (0–10 µg/ml). Bound antibody was detected using labelled anti-mouse IgG. Binding curves for the three antibodies were superimposed and showed strong binding to both human and mouse C7. The assays were repeated three times with comparable results. The error bars are standard errors of duplicates. (**D**) Classical pathway haemolytic assays (CH50). Nb62-r-mAb and control-r-mAb were tested for inhibition of complement-mediated lysis in human and mouse serum. Both r-mAb inhibited mouse and human serum-mediated haemolysis as effectively as the native parent mAb 73D1. The experiments were repeated three times with comparable results. The error bars are standard errors of duplicates. (**E**) Surface plasmon resonance (SPR) was used to determine the binding of Nb62-r-mAb and control-r-mAb to human and mouse C7. Human or mouse C7 was immobilized directly onto a CM5 sensor chip, and the relevant r-mAb or native mAb 73D1 was flowed over the chip. Sensorgrams were collected and dissociation constants (KD) calculated using the Langmuir 1:1 binding model (Table 1). Representative sensorgrams are shown with fitted data in black (*n* = 3).

Monoclonal antibodies (mAb) against C7 protein were produced by immunization of C7 deficient mice as described previously.^27^ The mAb 73D1, a strong blocker of mouse C7, was selected for generation of the recombinant mAb (r-mAb). The variable light and heavy chain of the mAb were sequenced (https://absoluteantibody.com/) and fused respectively to a mouse kappa light constant domain and mouse IgG2a framework, in which the D265A mutation had been introduced to disable FcγR binding.^33^ One heavy chain of the r-mAb was modified at the carboxy terminus to include a nanobody, Nb62, that binds to TfR, expressed on brain endothelium and shown to confer shuttle delivery to brain.^28,29^ A control non-brain penetrant r-mAb was generated by substituting an anti-GFP nanobody for Nb62. Both r-mAbs incorporated a 6-His tag and an ALFA tag for use in purification and detection, respectively. A cartoon of the control and brain-penetrant r-mAbs is shown (Fig. 1A). The r-mAbs were produced in-house by transient expression in Expi293F cells (A14527, ThermoFisher Scientific) for 5 days according to the manufacturer’s instructions. Briefly, plasmid DNA (1 μg/ml) and ExpiFectamine™293 reagent (A14524, ThermoFisher) were diluted with Opti-MEM™ reduced serum medium (ThermoFisher) and incubated for 5 min at RT. The plasmid DNAs were mixed in 1:1:1 ratio of the mAb light chain: heavy constant domain chain: heavy variable chain. The diluted ExpiFectamine™293 reagent was mixed with diluted plasmid DNA, incubated at RT for 20 min, and then slowly added with shaking to Expi293F cells (3 × 10^6^ cells/60 ml). Cells were then incubated in a humidified incubator (37°C, 5% CO_2_) on an orbital shaker. After 20 h, ExpiFectamine™293 Transfection Enhancers 1 and 2 (Cat. No. A14524, ThermoFisher) were added. Culture medium was harvested 5 days post-transfection and diluted 1:1 with PBS pH 7.4 prior to affinity purification of the r-mAb on a protein G column (Cat. No. 17-0405-01, Cytiva).

**Table 1:** Binding of Nb62-recombinant monoclonal antibody (r-mAb) and control-r-mAb to human and mouse C7 was measured using surface plasmon resonance

Protein	mAb	ka (1/Ms)	kd (1/s)	KD (M)
Mouse C7	Nb62-r-mAb	1.536 × 10^6^	6.910 × 10^−6^	4.499 × 10^−12^
	control-r-mAb	1.256 × 10^6^	1.350 × 10^−6^	1.075 × 10^−12^
	mAb 73D1	2.444 × 10^6^	1.621 × 10^−5^	6.630 × 10^−12^
Human C7	Nb62-r-mAb	9.556 × 10^5^	4.324 × 10^−4^	4.525 × 10^−10^
	control-r-mAb	4.703 × 10^5^	4.513 × 10^−4^	9.596 × 10^−10^
	mAb 73D1	1.041 × 10^6^	8.225 × 10^−4^	7.897 × 10^−10^

Human or mouse C7 was immobilized directly onto a CM5 sensor chip and the relevant r-mAb or native mAb 73D1 was flowed over the chip. Sensorgrams were collected and KDs calculated using the Langmuir 1:1 binding model (*n* = 3). Both the r-mAb and mAb 73D1 strongly bound mouse and human C7 (KDs ∼10^−12^, ∼10^−10^ respectively); the association constant (ka; on rate), dissociation constant (kd; off rate) and calculated KD are stated in the table.

### Characterizing purified r-mAb by SDS-PAGE and ELISA

The control and brain-penetrant r-mAb (5 µg) were resolved by sodium dodecyl sulfate–polyacrylamide gel electrophoresis (SDS-PAGE) under reducing and non-reducing conditions on 7.5% PAGE gels. Gels were stained with Coomassie Blue dye (Cat. No. NB4500078-1l, Generon). Direct ELISA was used to test binding of the r-mAb to human and mouse C7 immunoaffinity purified in-house as previously described.^27,34^ MaxiSorp (ThermoFisher) 96-well plates were coated with C7 (mouse or human, 0.5 µg/ml in bicarbonate buffer, pH 9.6) at 4°C overnight, blocked (30 min at 37°C) with 2% bovine serum albumin (BSA) in PBS and washed in PBS containing 0.05% Tween 20 (PBS-T). Dilutions of purified parent mAb or r-mAb, 1000–0 ng/ml [stock concentrations of all proteins measured using the Bicinchoninic Acid (BCA) assay (Cat. No. 23235, ThermoFisher)] in 0.2% BSA-PBS were added in triplicate to C7-coated wells and incubated for 1 h at 37°C. Wells were washed with PBS-T then incubated (1 h, 37°C) with secondary antibody [donkey anti-mouse-horseradish peroxidase (HRP); Cat. No. 715-035-150, Jackson ImmunoResearch]. After washing, plates were developed using *O*-phenylenediamine dihydrochloride (OPD, SigmaFastα™; Sigma-Aldrich) and the absorbance (492 nm) measured.

### Surface plasmon resonance analysis of native and recombinant mAb binding affinity to mouse and human C7

Surface plasmon resonance (SPR) binding analyses were carried out on a Biacore T200 instrument (Cytiva). All protein reagents used were of high purity and polished by size exclusion chromatography immediately before use to ensure the removal of aggregates. Human or mouse C7 was immobilized directly onto the CM5 sensor chip by amine coupling (Cat. No. 29-1496-03; Cytiva) to approximately 250 response units (RU). Control or brain-penetrant r-mAb or native mAb in HEPES-buffered saline (HBS; 10 mM HEPES, pH 7.4, 150 mM NaCl) containing 0.05% surfactant P20 (HBS-EP) was flowed over the immobilized C7 in a concentration series from 16 to 0.5 nM and interactions with the immobilized C7 were measured. For kinetic analysis, the flow rate was maintained at 30 μl/min and data were collected at 25°C. Data from a reference cell were subtracted to control for bulk refractive index changes. The Rmax was kept low and the flow rate high to eliminate mass transfer. Data were evaluated using BIACore Evaluation software (Cytiva).

### Haemolysis assays

The complement inhibitory activities of the native mAb and r-mAb in normal human serum (NHS) and normal male mouse serum (NMS) were investigated using classical pathway (CP) haemolysis assays (CH50). Sheep erythrocytes (ShE) were sensitized with rabbit anti-ShE antiserum (ShEA; Cat. No. ORLC25, Siemens Amboceptor; Cruinn Diagnostics), then suspended at 2% (vol:vol) in HBS containing Ca^2+^ and Mg^2+^. To measure CP activity in NMS, ShEA were additionally incubated with mouse anti-rabbit IgG (Cat. No. 3123; Invitrogen; 25 µg/ml) for 30 min at 37°C before washing and re-suspending in HBS.^32,35^ Serum dilutions for each species were selected in preliminary experiments to give near-complete haemolysis in the CP assay in the absence of test mAb, typically 2.5% for NHS and 25% for NMS (using the double-sensitized cells). A serial dilution series of native mAb or r-mAb (100–0 µg/ml) was prepared in HBS and aliquoted in triplicate into a 96-well round-bottomed plate at 50 µl/well, then serum at the appropriate dilution and 2% ShEA (50 µl/well of each; double-sensitized for mouse as above) added. Plates were incubated at 37°C for 30 min, centrifuged and haemoglobin in the supernatant was measured by absorbance at 405 nm. For each assay, percentage lysis was calculated according to: %Lysis = Absorbance (Abs) sample − Abs background)/(Abs max − Abs background) × 100%. GraphPad Prism (v. 9.0) was used for data analysis.

### Preparation of total brain homogenate, brain tissue-bound protein and peripheral organ lysates

Total brain homogenate (TBH) was prepared by homogenizing individual snap-frozen brain hemispheres in 0.3 ml of ice-cold radio-immunoprecipitation assay (RIPA) buffer (Cat. No. R0278-50ML, Sigma Aldrich) supplemented with 1 × EDTA-free protease inhibitor (Cat. No. 4693159001, Roche cOmplete mini EDTA-free, Sigma Aldrich) for 5 min on ice using a motorized homogenizer (431-0100, VWR). Lysed samples were centrifuged at 13 000 rpm for 20 min at 4°C and the supernatant (TBH) collected. The remaining pellet was further processed as described previously^36^ to prepare the brain tissue-bound protein (TBP) fraction containing aggregated Aβ and tissue-fixed complement proteins. Briefly, 0.3 ml 8 M Guanidine-HCl (Cat. No. 24115, ThermoFisher) was added (final concentration of Guanidine-HCl in the sample was 5 M), the pellets homogenized on ice as described above, incubated on ice for 30 min then centrifuged; the resultant supernatant (TBP) was collected for ELISA measurements. Total protein concentration in the extracts was measured using the Pierce Micro BCA Protein Assay Kit (Cat. No. 23235, ThermoFisher). TBH and TBP samples were adjusted to 1 mg/ml, aliquoted and frozen immediately after preparation, stored at −80^°^C until testing and not subjected to freeze-thaw.

Lysates of other peripheral organs (liver, spleen, kidney, heart, lung, eye and muscle) were made to explore tissue distribution of the fluorescent proteins. Organs were harvested, diced and homogenized in PBS (3:1 v/w ratio) containing 1% Triton X-100 supplemented with protease inhibitors (cOmplete, Sigma) using a motorized homogenizer as described above and incubated for 30 min on ice. Lysates were spun down at 13 000 rpm for 20 min at 4°C, the supernatant collected, protein measured, standardized as above and stored in aliquots at −80°C prior to analysis.

### Detection of recombinant monoclonal antibodies in brain and peripheral organ lysates and serum

Levels of control r-mAb or Nb62 r-mAb in brain lysates (TBH), organ lysates and serum from the treated mice were measured using an ELISA. TBH and organ lysates were used neat or diluted 1:2 in PBS; serum was diluted 1:500 in PBS. The test samples were incubated on nickel-coated plates (ThermoFisher, Cat. No. 15142) for 2 h at RT to capture the r-mAb via the His tag, wells washed in PBS-T then HRP-labelled detection mAb (1G5 anti-ALFA tag-HRP; Cat. No. N1502-HRP-SY, 2BScientific) added (1:3000 in PBS; 1 h at RT). Plates were washed, developed using OPD and absorbance (492 nm) measured. Standard curves were generated using the respective r-mAbs (500–0 ng/ml) enabling quantification in the samples. The intra- and inter-assay precision was calculated (%CV; <5%).

The presence of r-mAb in TBH from the treated mice was confirmed using western blotting. TBH were diluted 1:2 in PBS, resolved on SDS-PAGE under non-reducing conditions and electrophoretically transferred to 0.45 μm nitrocellulose membranes (GE Healthcare); membranes were blocked with 5% BSA in PBS-T, washed in PBS-T, incubated for 1 h at RT with HRP conjugated anti-ALFA tag antibody (2.5 μg/ml; Nanotag Biotechnologies, Cat. No. N1501-HRP) in 5% BSA PBS-T, washed, developed with enhanced chemiluminescence (GE Healthcare) and visualized by autoradiography.

### Detection of IL-1α and IL-1β by ELISA in TBH

Levels of IL-1α and IL-1β in TBH were measured using commercial ELISA kits (respectively, Cat. No. 88-5019-22, Invitrogen; Cat. No. DY401-05, R&D Systems) following the manufacturer's instructions. Briefly, wells were coated with capture antibody (1:250 in PBS, 100 µl/well) overnight at RT, washed, blocked and then incubated with 100 µl of TBH (1:2) or standards (serial dilutions: 500–0 pg/ml). After washing, the detection antibody was added, followed by streptavidin-HRP. The assay was developed using TMB, absorbance at 450 nm was recorded, and the IL-1α or IL-1β levels in the TBH samples were read from the standard curve and expressed as pg/mg total protein.

### Detection of complement activation products in brain and serum by sandwich ELISA

The C3 fragment (C3b/iC3b/C3c) and terminal complement complex (TCC; fluid phase marker of terminal pathway activation) assays were performed as described previously.^38^ For TCC detection, Maxisorp (ThermoFisher) 96-well plates were coated with rabbit anti-rat/mouse C9 IgG (10 µg/ml in bicarbonate buffer pH 9.6) at 4°C overnight; wells were blocked (30 min at 37°C) with 3% BSA in PBS-T and washed in PBS-T. TBH or TBP were diluted 1:100, sera 1:20 in 0.3% BSA PBS-T containing 10 mM EDTA (PBS-T-EDTA), added in duplicate to ELISA wells and incubated overnight at 4°C. Wells were washed with PBS-T, then HRP-labelled detection mAb 12C3 anti-mouse TCC-neo was added at 5 µg/ml in PBS-T-EDTA and incubated for 1.5 h at RT. Plates were washed, developed using OPD and the absorbance (492 nm) was measured. Standard curves were generated using serial dilutions of in-house activated normal male mouse serum (Act-NMS) prepared as described previously.^27^ Results were expressed as units (U) per mg total protein. Two Act-NMS samples (1 in 800) were included as inter-assay controls across all plates and assays to calculate intra- and inter-assay precision (%CV; <10%).

To measure mouse C3b/iC3b/C3c, plates were coated with 2/11 mAb anti-mouse C3b/iC3b/C3c (5 µg/ml, HM1065, Hycult Biotech), blocked in 3% BSA-PBS-T and washed in PBS-T. TBH or TBP were diluted 1:800, sera 1:20 000 in 0.3% BSA-PBS-T-EDTA, added in duplicate to ELISA wells, incubated overnight at 4°C, washed and bound C3 fragments detected using in-house HRP-labelled rabbit anti-human C3 (cross-reactive with mouse), 1:500 in 0.3% BSA-PBS-T-EDTA for 1.5 h at RT. Plates were washed, assays developed with OPD and absorbance measured as above. Standard curves were generated using Act-NMS from a starting dilution of 1:5000 in 0.3% BSA-PBST-EDTA in duplicate and the results expressed as U/mg total protein. The intra- and inter-assay precision was calculated from internal standards as above (%CV; <10%).

### Detection of amyloid load by ELISA and immunofluorescence in APP^NL-G-F^ mouse brain

To measure human Aβ_42_ levels in TBP extracts of APP^NL-G-F^ brains, a commercial assay (KHB3442, ThermoFisher) was used according to the manufacturer’s protocol. In brief, TBP samples (1:4000) or standard (2000–0 pg/ml) were added in duplicate (50 µl/well) into the capture antibody-coated wells, incubated, washed, then biotinylated detection antibody (50 µl/well) added and incubated 3 h at RT with shaking. After washing, 100 µl of Streptavidin-HRP was added (1:100), incubated for 30 min at RT, washed, developed with TMB and absorbance (450 nm) measured. Concentrations of Aβ_42_ in the TBP were calculated from the standard curve and the results expressed as pg/mg total protein.

For assessment of amyloid plaque load, fixed brains were sectioned using a Leica VT1200S vibratome (Leica Biosystems); free-floating fixed brain sections were incubated in 100 µM X-34 (Sigma, SML1954) in 40% ethanol, 60% dH_2_O, 0.05 M NaOH for 20 min at RT, washed in 40% ethanol, 60% dH_2_O for 5 min and then twice in PBS-T before mounting in FluorSave (Millipore).

To characterize plaque load and type, immunofluorescence was performed. Fixed free-floating sections were incubated in 70% formic acid for 15 min at RT on a shaker, washed in dH_2_O for 2 min at RT, and then in PBS for 20 min at RT. Sections were incubated in blocking solution [5% normal goat serum (Vector labs, S-1000-20) in PBS-T] for 2 h at RT, then with anti-Aβ antibodies (residues 17-24, 4G8-Alexa Fluor 488; residues 1-16, 6E10-Alexa Fluor 594; BioLegend Cat. No. 800 714 and 803 019, respectively; both at 1:250 in blocking solution) for 48 h. Sections were washed in PBS-T, incubated in 1% Sudan black in 70% ethanol for 20 min at RT, then washed sequentially in 70% ethanol, 50% ethanol and PBS. Sections were counterstained in DAPI and mounted in Vectasheild vibrance (VectorLab, Cat. No. H1700).

Images from the hippocampus CA1 region and overlying cortex were taken on a Leica SP8 Lightning confocal microscope (10× objective; 60-µm stack with *z*-axis interval 8.5 µm; image size 1550 µm^2^). Excitation was set at 405 nm (HyD laser power 5%; gain 10%) with the detection window set at 450−600 nm. Plaque images were batch analysed in Imaris (version 10.0, Bitplane) by combining them into a time series and isolating the relevant regions using the Surface and Masking function. Images were then normalized across the batch using the Imaris XTensions ‘Normalise Time Points’ tool, followed by automatic plaque quantification using the Surface function.

### Dendritic spine labelling, imaging and analyses

For the measurement of dendritic spines, we used unbiased ballistic labelling by DiOlistics on fixed 60 µm free-floating fixed brain sections. Ten brain hemisphere sections per mouse covering the dorsal hippocampal field were transferred to histology slides and subjected to neuronal DiOlistic labelling as previously described.^37,38^ Tungsten microcarrier particles coated with 1,1′-dioctadecyl-3,3,3′,3′-tetramethylindocarbocyanine perchlorate (DiI; Life Technologies) were fired at a pressure of 80 psi onto tissue sections through an inverted cell culture insert (8.0 µm; BD Falcon, BD Biosciences). Dye was allowed to diffuse by incubating sections at RT for 1 h in PBS; sections were fixed with 4% PFA and then mounted in FluorSave (Millipore). Dye-labelled CA1 hippocampal neurons were confocally imaged using a Leica SP8 Lightning confocal microscope and deconvolved using Leica Lightning Deconvolution (63× objective; *z*-axis interval 0.189 µm). Labelled dendritic spines were captured from CA1 secondary apical dendrites within the CA1 stratum radiatum field. Images were blind analysed using the Imaris FilamentTracer module (version 9.2, Bitplane) and quantified as one batch to minimize operator bias. Spine subtypes (stubby, mushroom, thin) were classified based on predefined morphology using the SpineClassifer MATLAB extension.

For assessment of synaptic puncta, free-floating brain sections were subjected to epitope retrieval, permeabilized in Triton X-100 (1% v/v in PBS), blocked (5% NGS in PBS-T) and incubated with primary antibodies against PSD95 (Abcam, Cat. No. ab18258, rabbit) and Bassoon (Synaptic Systems, Cat. No. 141004, guinea pig) at 1:500 in blocking buffer for 48 h at 4°C. Species-specific Alexa Fluor Plus secondary antibodies (Invitrogen, 1:500) were then added for 2 h at RT. Endogenous autofluorescence was quenched with Sudan Black, and nuclei were stained with DAPI. Sections were mounted in Vectashield Mounting Medium and stored at 4°C in the dark until imaged using a Leica SP8 Lightning confocal microscope. Sections from four mice per group were imaged, *z*-stacks (z = 0.6 µm, interval 0.12 µm) generated and 12 fields per mouse imaged and analysed in Imaris by combining maximum projections into a ‘time series’, allowing batch analysis using the Imaris XTensions ‘Normalise Time Points’ tool to standardize the image intensities, followed by automated quantification using the Imaris Surface function with a predefined threshold cut-off relative to the background signal.

### Brain penetrance of recombinant mAbs and impact on complement activation and neurodegeneration

To test the capacity of the r-mAbs to access the brain following systemic administration, we used C7-deficient (C7−/−) mice; this choice eliminated the complication of ligand (C7) binding to the r-mAb in the periphery or brain. C7−/− mice (aged 4–6 weeks) were administered either Nb-62-r-mAb or control-r-mAb by intraperitoneal (i.p.) injection (0.1 mg in PBS, 4 mg/kg dose), then sacrificed at 2, 4 and 24 h post-injection (four mice per time point for each agent), blood harvested for serum, perfused intracardially with PBS then brains harvested and snap frozen. Serum and TBH were generated, and levels of r-mAb measured by ELISA as described above.

To test the impact of the r-mAbs on complement activation and AD pathology, male APP^NL-G-F^ mice aged 5–6 months (‘young mice’; *n* = 15) or 11–13 months (‘old mice’; *n* = 11) were first injected subcutaneously (s.c.) with a saturating dose of mAb 73D1 (40 mg/kg in PBS) to swamp the peripheral C7; 2 h later, either Nb62-r-mAb or control-r-mAb were delivered by intraperitoneal injection at a dose of 4 mg/kg to ‘young mice’ (Nb62-r-mAb, *n* = 8; control-r-mAb, *n* = 7) and ‘old mice’ (Nb62-r-mAb, *n* = 6; control-r-mAb, *n* = 5). A second set of injections, 73D1 (40 mg/kg s.c.) followed by the relevant r-mAb (2 mg/kg i.p., for all mice), was given on Day 3. Mice were tail bled on Days 0, 3 and 7 prior to any injections, and serum was harvested and tested for haemolytic activity as described previously.^27^ Mice were sacrificed on Day 7, PBS perfused, brains harvested and spilt sagittally; one half was snap frozen for preparation of TBH and TBP extracts for measurement of levels of agent, complement activation markers and Aβ, and the other half fixed in 1.5% PFA for measurement of spine density by DiOlistics and amyloid plaque load by immunohistochemistry.

In a separate experiment, APP^NL-G-F^ mice aged 6–9 months were randomly assigned to two groups of 12. All mice received anti-C7 mAb twice weekly (s.c.), while Group 1 additionally received control-r-mAb and Group 2 Nb62-r-mAb with dosing and schedules as for the 7-day study but extended for 13 weeks. Mice were tail-bled at intervals, and serum was harvested to measure complement activity. Prior to sacrifice, mice were subjected to behavioural testing using burrowing, open field and novel object recognition tests as described below. Mice were sacrificed on Day 91, PBS perfused, brains harvested and spilt sagittally; one half was snap frozen for preparation of TBH and TBP extracts for measurement of levels of r-Mab, complement activation markers, inflammatory cytokines and Aβ, and the other half fixed in 1.5% PFA for measurement of spine density by DiOlistics and plaque load by immunohistochemistry.

### Burrowing, Open Field and Novel Object Recognition behavioural tests

All tests were performed with randomized mice, and the operator was blinded to group assignments. To minimize stress, animals were acclimatized to the experimental room in their home cages for a week prior to testing. All experiments were conducted in the same dimly lit room illuminated with red lights to ensure consistency. For assessment of burrowing activity, the mouse was placed overnight in a fresh cage (90 cm × 60 cm × 120 cm) containing a tube filled with 200 g of pea gravel. The next morning, the remaining pea gravel in the tube was weighed and the percentage burrowed was calculated.^39^

For the Open Field test, mice were placed in the centre of an opaque box (40 × 40 × 40 cm). Every experiment commenced with the mouse positioned in the centre; movements were recorded using a fixed overhead camera for 7 min. The percentage of time spent in a marked central zone was determined by reviewing the videos and measuring the time spent in both peripheral and central zones.^40^

The Novel Object Recognition (NOR) test was conducted in the same box but with visual cues (star and cross) on the walls. To acclimatize, mice were placed in the box for 10 min on two consecutive days without object cues. On the third day (Day 1; training phase), two identical objects (A and A′) were introduced for 10 min, with exploration behaviour recorded on video and time spent examining each object noted. The following day (Day 2; test phase), mice were presented with two objects: one from the training phase (A) and a novel object (B) for 10 min; exploration behaviour was recorded and analysed as before.^41^ Cages and objects were meticulously cleaned between experiments to eliminate scent clues and ensure unbiased results.

### Statistics

Statistical calculations were performed with GraphPad Prism Software version 9.4.1 (San Diego, California, USA). Data are presented as mean ± standard deviation. The statistical significance of differences between two groups was obtained using the unpaired t-test, and for multiple groups, one-way ANOVA was used after testing for normality. All animal studies survived to the planned end point, enabling all data points to be included in the analysis. Behavioural tests and all histopathologic analysis and quantification, including DiOlistics, were performed blinded to experimental groups.

## Results

### The recombinant monoclonal antibodies bind C7 and inhibit complement *in vitro*

The control-r-mAb and Nb62-r-mAb derived from the 73D1 mAb were expressed in Expi293 cells and aseptically purified to homogeneity on protein G; each r-mAb ran as a single band of ∼150 kDa (intact r-mAb) in non-reduced SDS-PAGE and three bands under reducing conditions; ∼55 kDa, ∼70 kDa (r-mAb heavy chains; one with the nanobody attached) and ∼25 kDa (r-mAb light chain) (Fig. 1A and B). Direct ELISAs showed that both r-mAb and the native mAb 73D1 recognized human and mouse C7 (Fig. 1C). The capacity of r-mAb to inhibit complement was compared with that of the parent mAb 73D1; both r-mAb inhibited complement activity in human and mouse serum with the same efficiency as the benchmark 73D1 mAb (Fig. 1D). Strong binding of r-mAb and 73D1 to mouse and human C7 was confirmed by SPR analysis on immobilized mouse or human C7 (Fig. 1E). The r-mAbs displayed very slow off-rates as previously reported for clone 73D1, an efficient inhibitor of complement *in vivo.*^29^ The calculated dissociation constants (KDs; not true Kds because of antibody avidity but valid comparators) were similar for the two r-mAb and the parent 73D1 mAb, demonstrating that the cloning process had not adversely impacted the binding of r-mAb to C7 ligand (Fig. 1F).

### The Nb62-r-mAb penetrates mouse brain *in vivo*

**Figure 2:**
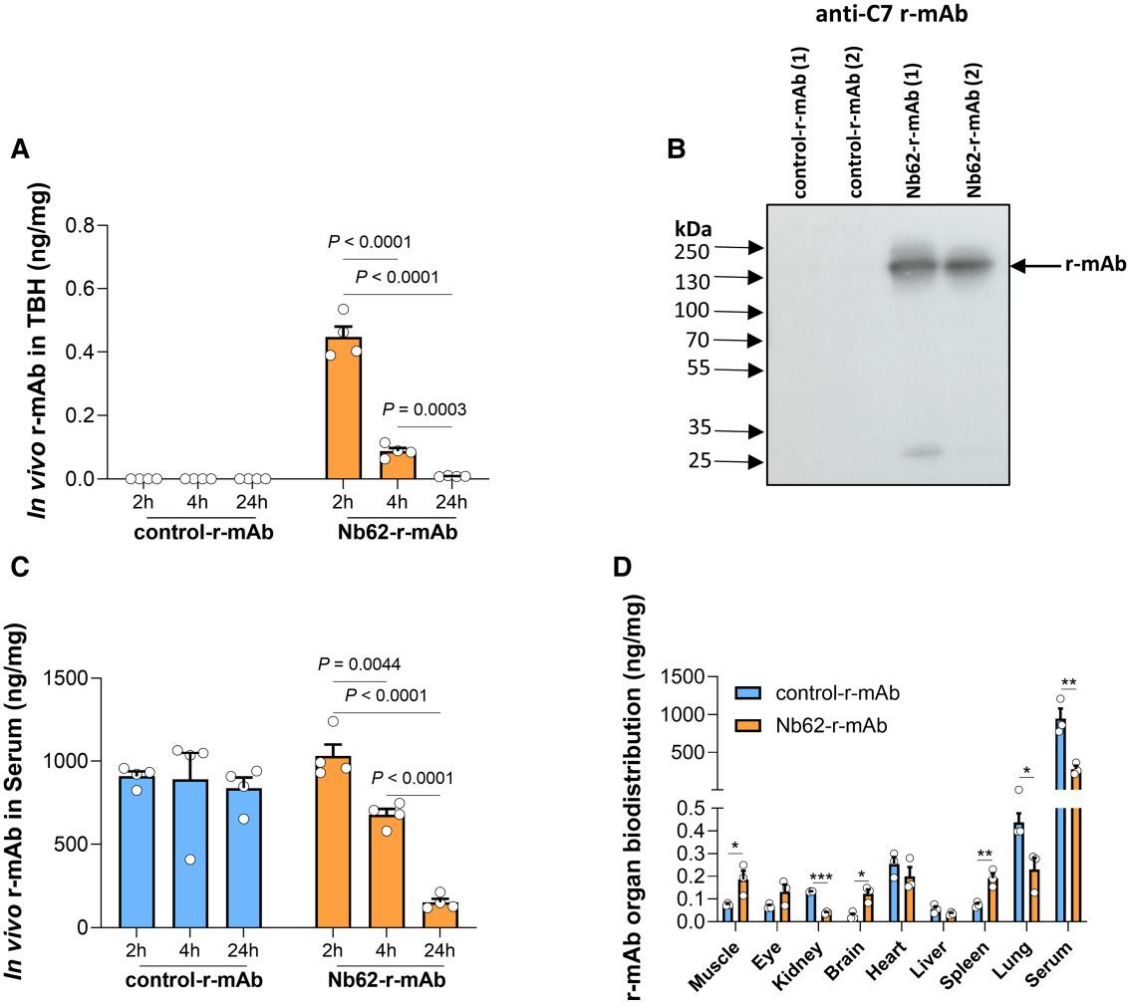
***In vivo* testing of the recombinant monoclonal antibodies (r-mAbs) for delivery to the brain.** Entry of Nb62-r-mAb and control-r-mAb into brain was tested in 8-week-old C7-deficient mice. (**A**) r-mAb in total brain homogenate (TBH) was detected by sandwich ELISA and expressed as ng/mg protein. Nb62-r-mAb was detected in TBH at 2, 4 and 24 h time points, whereas control-r-mAb was not detected at any time point. (**B**) Western blotting confirmed the presence of Nb62-r-mAb but not control-r-Mab in TBH. (**C**) r-mAb levels in serum were measured by ELISA; both Nb62-r-mAb and control-r-mAb were detectable at each time point but significantly less Nb62-r-mAb was detected at 4 and 24 h (*P* = 0.0044 and *P* < 0.0001, respectively), suggesting rapid clearance. (**D**) Distribution of the r-mAbs in other organs at 2 h was tested in tissue lysates using ELISA; distribution patterns were different with the Nb62-r-mAb higher in muscle, brain and eye. *P < 0.05, **P < 0.01. All assays were repeated three times with comparable results. The error bars are standard errors of triplicates. Unpaired two-tailed *t*-test was used for the group comparison. Error bars correspond to the standard error of the mean.

To test BBB penetrance *in vivo*, C7−/− mice (4–6 weeks) were treated with a single dose of Nb62-r-mAb or control-r-mAb (4 mg/kg, i.p.); groups of mice were sacrificed at 2, 4 and 24 h (*n* = 4 for each r-mAb at each time point), blood and brains harvested. Nb62-r-mAb was detected by ELISA in brain homogenates (TBH) at each time point, albeit with sharply reducing levels with time (2 h, 0.448 ng/mg; 4 h, 0.087 ng/mg; 8 h, 0.008 ng/mg); in contrast, control-r-mAb was not detected in TBH at any time point, demonstrating that levels in brain did not reach the assay detection limit (<0.005 ng/mg) at any time point (Fig. 2A). Presence of Nb62-r-mAb in TBH was confirmed by western blotting at the 2 h time point; no control-r-mAb was detected in TBH westerns at this time point (Fig. 2B). Both proteins were strongly detected in serum across the study duration; however, while levels of the control-r-mAb were unchanged over the 24 h period, serum levels of Nb62-r-mAb fell sharply between 2 and 24 h, (Fig. 2C). These data suggest that the TfR-targeting Nb62 nanobody mediates enhanced clearance through binding to TfR, expressed ubiquitously in peripheral organs. To test this, C7−/− mice were given a single IP dose of Nb62-r-mAb or control-r-mAb (4 mg/kg; four per group), sacrificed at 4 h and r-mAb concentrations in various organs measured by ELISA (Fig. 2D). Nb62-r-mAb was present at significantly higher levels compared to control-r-mAb in brain, muscle, spleen and eye of treated animals at 4 h, confirming enhanced peripheral uptake. In contrast, significantly more control-r-mAb was detected in kidney and lung.

### C7 inhibition reduces complement activation, synapse loss and amyloid-β levels in AD mouse brain

**Figure 3:**
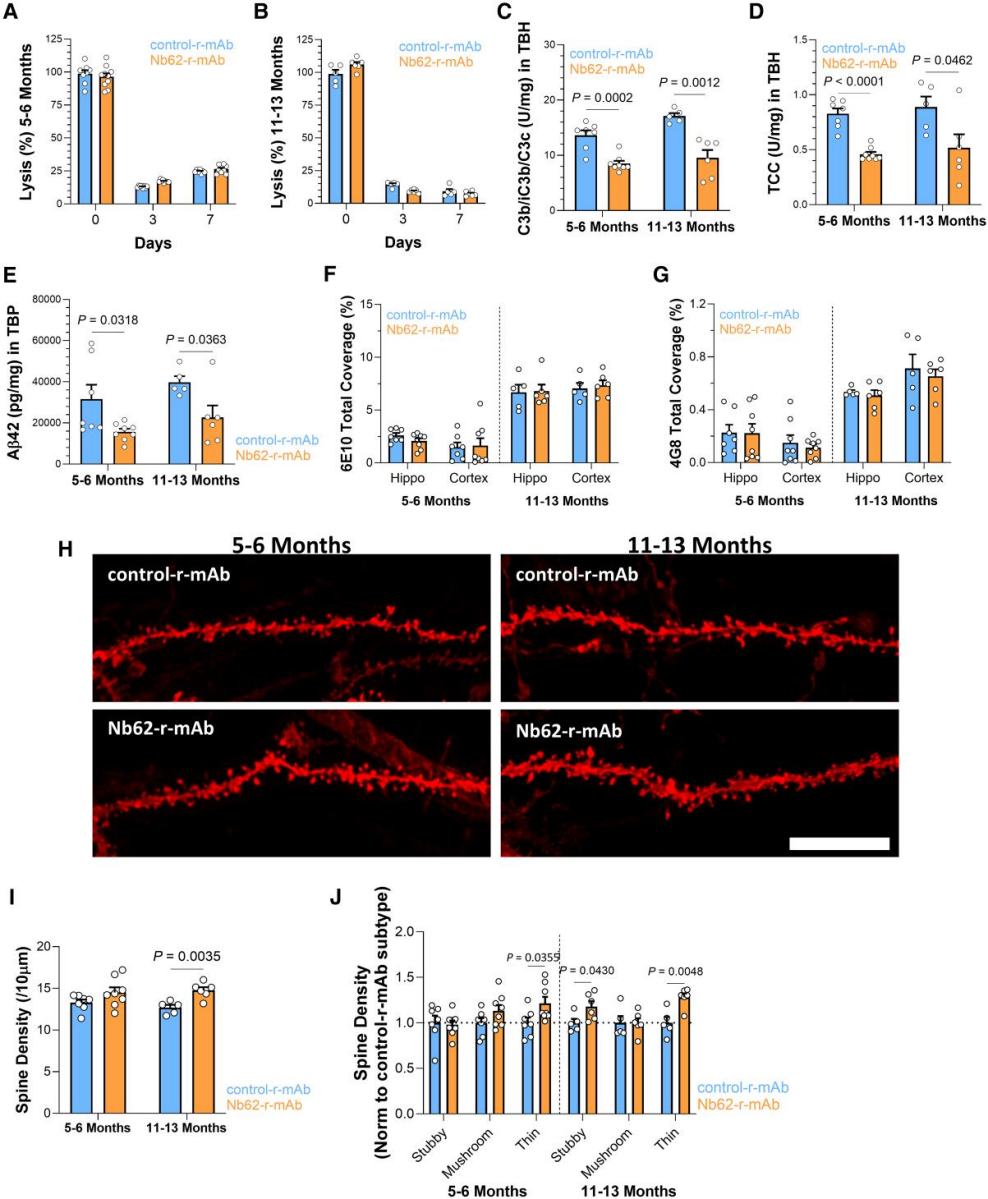
**Impact of 1-week treatment with Nb62-recombinant monoclonal antibody (r-mAb) on brain parameters in APP^NL-G-F^ mice.** APP^NL-G-F^ mice were treated with anti-C7 mAb plus either Nb62-r-mAb or control-r-mAb over 7 days; systemic inhibition of complement, inhibition of complement activation in brain, brain inflammation, and neurodegeneration were assessed. (**A** and **B**) Classical pathway haemolysis (CH50) assays confirm that systemic complement was inhibited in APP^NL-G-F^ mice aged 5–6 months (control-r-mAb: *n* = 7, Nb62-r-mAb: *n* = 8) or 11–13 months (control-r-mAb: *n* = 5, Nb62-r-mAb: *n* = 6) over 1 week of systemic administration of 73D1 mAb and either r-mAb. (**C** and **D**) Sandwich ELISAs detecting mouse C3 fragments (C3b/iC3b/C3c; **C**) and terminal complement complex (TCC; **D**) in total brain homogenate (TBH). Both C3 fragments and TCC levels were significantly lower in Nb62-r-mAb-treated mice at either age. Error bars are standard errors for each dataset. Groups were compared using an unpaired two-tailed *t*-test. (**E**) Sandwich ELISA to measure levels of amyloid-β (Aβ) in tissue bound protein (TBP) demonstrating significantly decreased Aβ in TBP of Nb62-r-mAb-treated APP^NL-G-F^ mice compared to controls in both age sets. (**F** and **G**) Immunostaining of Aβ plaques in hippocampus and cortex using anti-Aβ antibodies: 6E10 (**F**) and 4G8 (**G**) showed no significant difference in plaque coverage between Nb62-r-mAb- and control-r-mAb-treated APP^NL-G-F^ mice at either age. (**H**) Representative confocal images of DiOIistics-labelled CA1 hippocampal dendritic segments in 5–6-month-old and 11–13-month-old APP^NL-G-F^ mice treated with Nb62-r-mAb or control-r-mAb. Scale bar = 5 µm. (**I** and **J**) DiOIistics-labelled dendritic spines were analysed in prefixed coronal brain slices. Overall spine density, analysed from dendritic segments of at least 30 µm, was higher in Nb62-r-mAb-treated mice compared to controls at both ages, significant only in the 11–13-month set. Analysis of spine subtypes showed that the number of thin spines was significantly increased in Nb62-r-mAb-treated groups in both age sets. Unpaired two-tailed *t*-test was used to compare spine densities between groups. Error bars correspond to the standard error of the mean.

**Figure 4:**
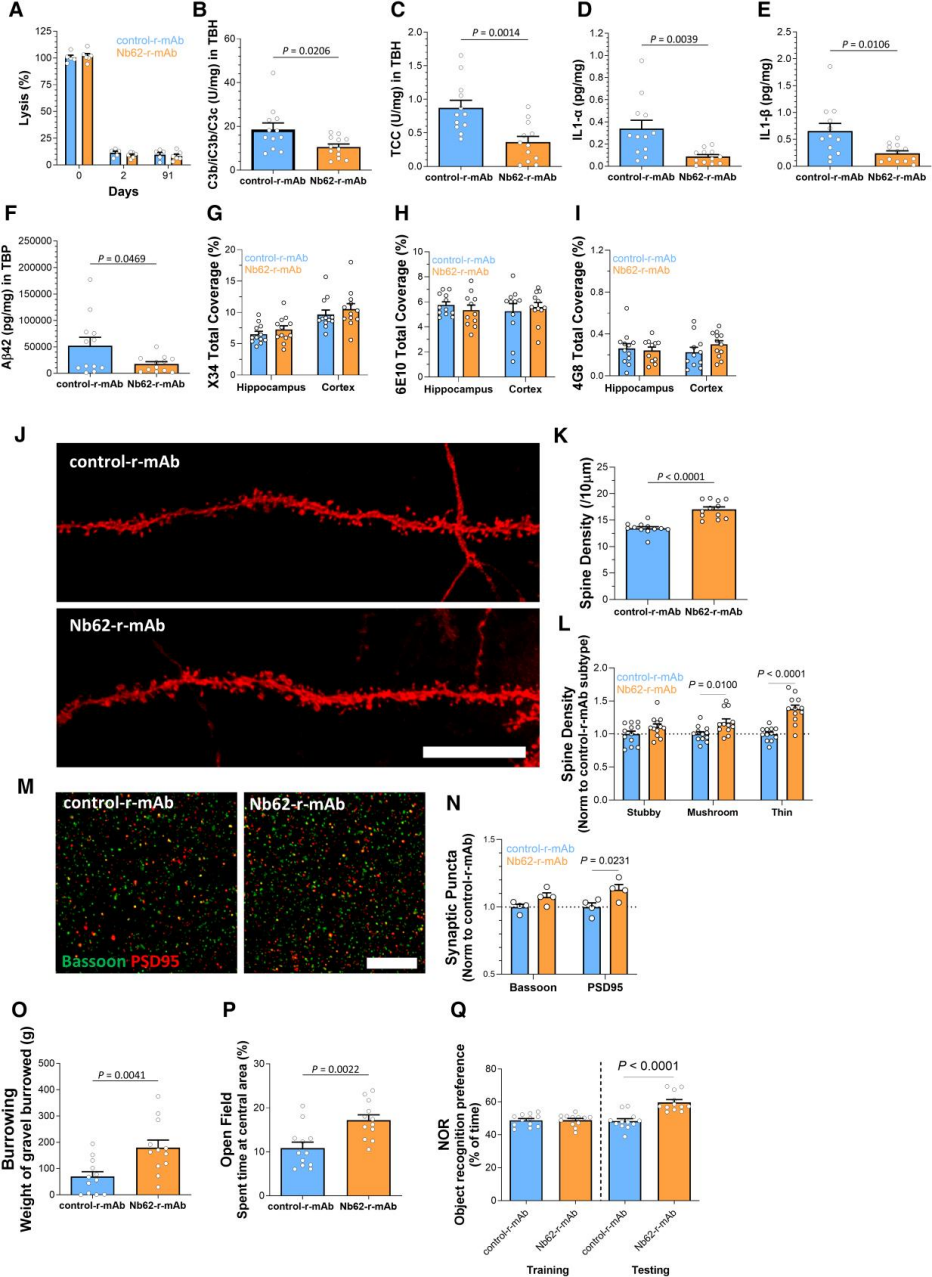
**Impact of 3** **months of treatment with Nb62-r-mAb on brain parameters in APP^NL-G-F^ mice.** APP^NL-G-F^ mice were treated with anti-C7 mAb plus either Nb62-r-mAb (*n* = 12) or control-r-mAb (*n* = 12) over 3 months; systemic complement activity, complement activation in brain, brain inflammation, neurodegeneration and cognition were assessed. (**A**) Classical pathway haemolytic assays (CH50) demonstrated that systemic complement was inhibited in the Nb62-r-mAb and control-r-mAb-treated mice over the 3-month time course. (**B** and **C**) Levels of C3 fragments (C3b/iC3b/C3c; **B**) and terminal complement complex (TCC; **C**) in the total brain homogenate (TBH) were significantly reduced at end point in Nb62-r-mAb-treated APP^NL-G-F^ mice compared to controls. (**D** and **E**) Levels of cytokines: IL-1α and IL1-β in TBH at end point were significantly decreased in Nb62-r-mAb-treated APP^NL-G-F^ mice compared to controls. (**F**) Levels of amyloid-β (Aβ) in tissue bound protein (TBP) were significantly decreased at end point in Nb62-r-mAb-treated APP^NL-G-F^ mice compared to controls. (**G**–**I**) Aβ plaques in hippocampus and cortex were either stained with the plaque stain X34 (**G**) or immunostained with anti-Aβ antibodies 6E10 (**H**) and 4G8 (**I**); analysis revealed no significant differences in plaque coverage with any of these stains at end point between the Nb62-r-mAb- and control-r-mAb-treated APP^NL-G-F^ mice. (**J**) Representative confocal images of DiOIistics labelled CA1 hippocampal dendritic segments from APP^NL-G-F^ mice treated with Nb62-r-mAb or control-r-mAb. Scale bar = 5 µm. (**K** and **L**) Quantification of DiOIistics-labelled dendritic spines in prefixed coronal brain slices. (**K**) APP^NL-G-F^ mice treated with Nb62-r-mAb showed significantly increased overall spine density compared to control-r-mAb-treated mice. Analysis of spine subtypes showed that the numbers of thin and mushroom spines were significantly increased in Nb62-r-mAb-treated groups, most significantly for thin spines. (**M**) Representative images of Bassoon (green) and PSD95 (red) immunoreactive synaptic puncta in the stratum radium of Nb62-r-mAb- and control-r-mAb-treated APP^NL-G-F^ mice at end point; scale bar = 5 µm. (**N**) Synaptic puncta stained with Bassoon or PSD95 were quantified (region of interest, 20 µm × 20 µm, 12 per mouse) using Imaris Spot function; puncta were increased in Nb62-r-mAb-treated mice compared with controls for both stains but significantly only for PSD95. (**O**–**Q**) Comparison of Nb62-r-mAb-treated and control-r-mAb-treated APP^NL-G-F^ mice in behavioural tests. (**O**) In the burrowing test, Nb62-r-mAb-treated mice burrowed significantly more of the gravel compared to controls. (**P**) In the Open Field (OF) test, Nb62-r-mAb mice spent significantly more time exploring the central area of the box. (**Q**) In the Novel Object Recognition (NOR) test, Nb62-r-mAb-treated mice spent significantly more time exploring the novel object. Each point represents one animal in these analyses. For all quantitative analyses, an unpaired two-tailed *t*-test was used to compare the two groups. Error bars correspond to standard error of the mean, and *P*-values are included where appropriate.

APP*^NL-G-F^* mice, either 5–6 months (young) or 11–13 months (old), were treated on Days 0 and 3 with the parent mAb delivered subcutaneously to swamp systemic C7, followed 2 h later by Nb62-r-mAb or control-r-mAb delivered IP. Complement haemolytic activity in serum was reduced to background levels throughout the experiment, confirming that no free C7 remained in the circulation (Fig. 3A and B). Mice were sacrificed on Day 7, blood collected and perfused brains harvested and split sagittally. Brain tissue extracts (TBH, TBP) were prepared from one half and assayed in sandwich ELISAs for r-mAb levels, complement activation products and Aβ_42_ levels; the other half was reserved for imaging. Neither Nb62-r-mAb nor control-r-mAb were detected in TBH at Day 7 (data not shown), which was expected given the rapid clearance observed in the preliminary study and limited brain penetration, respectively. Despite this, levels of complement activation products, both C3 fragments and TCC, were significantly reduced in TBH from Nb62-r-mAb treated mice compared to controls in both young and old groups (C3 fragments; *P* = 0.0002 and *P* = 0.0012, respectively; TCC; *P* < 0.0001, *P* = 0.0462; Fig. 3C and D). Aβ_42_ levels in TBP samples were reduced significantly in Nb62-r-mAb treated young and old APP^NL-G-F^ mice compared to control-r-mAb (*P* = 0.0318 and *P* = 0.0363, respectively; Fig. 3E). Amyloid plaque coverage, assessed in cortex and hippocampus by staining with anti-Aβ antibody 6E10 (versus residues 4–10; Fig. 3F) or 4G8 (versus residues 18–23; Fig. 3G), was not different between Nb62-r-mAb and control-r-mAb treated mice in either site or age group.

To test the impact of 7-day treatment with Nb62-r-mAb on neurodegeneration, synapse loss was assessed using DiOlistics on fixed brain sections; representative images are shown in Fig. 3H. Spine numbers from CA1 hippocampal neurons (pooled dendrites per treatment mouse; average of at least 10 dendritic segments per mouse) and morphological subtypes were automatically quantified. In both young and old APP^NL-G-F^ mice treated with Nb62-r-mAb, overall spine density was higher compared to control-r-mAb treated age-matched APP^NL-G-F^ mice, significant in the old group (*P* = 0.0035; Fig. 3I). Comparison of different morphological spine subtypes showed that thin spine density was significantly different between Nb62-r-mAb and control-r-mAb treated mice in young (*P* = 0.0355) and old (*P* = 0.0048) groups (Fig. 3J).

### Prolonged C7 inhibition reduces pathology and improves cognition in Alzheimer’s disease mice

Male APP^NL-G-F^ mice aged between 6 and 9 months were randomized into two groups of 12; all mice received anti-C7 mAb twice weekly (SC) to block systemic C7, while Group 1 additionally received control-r-mAb and group 2 Nb62-r-mAb for 13 weeks with dosing and schedules as for the short treatment study. Systemic complement activity was completely inhibited throughout the time course (Fig. 4A). Mice were subjected to behavioural testing at 13 weeks, then sacrificed, brains harvested, and parameters of complement activation and pathology measured in lysates and sections, as for the short treatment study. Complement activation products in TBH were significantly reduced in Nb62-r-mAb treated APP^NL-G-F^ mice compared to control-r-mAb (C3 fragments, *P* = 0.0206; TCC, *P* = 0.0014; Fig. 4B and C), demonstrating target engagement by Nb62-r-mAb. To investigate the impact on neuroinflammation, the pro-inflammatory cytokines IL1-α and IL-1β were measured in TBH; both were significantly reduced in Nb62-r-mAb treated mice compared to controls (*P* = 0.0039, *P* = 0.0106; Fig. 4D and E). Levels of Aβ_42_ measured in TBP were markedly reduced in Nb62-r-mAb treated mice compared to controls (*P* = 0.0469; Fig. 4F).

The impact of Nb62-r-mAb administration on amyloid plaque coverage was assessed by staining with the well-characterized plaque marker X34 and anti-Aβ antibodies 6E10 and 4G8; per cent coverage, plaque number and average plaque size in hippocampus and cortex were automatically calculated in IMARIS. Although Aβ antibody (6E10 and 4G8) staining in hippocampus was lower in Nb62-r-mAb treated mice compared to controls, there were no significant differences between Nb62-r-mAb and control-r-mAb groups in any of these markers of plaque load in either location (Fig. 4G–I).

Dendritic spines were measured using DiOlistics on fixed brain slices as described in the short treatment study; spine numbers (from at least 10 pooled dendrites per treatment mouse) and morphological subtypes were quantified from CA1 hippocampal neurons. Representative images from control-r-mAb and Nb62-r-mAb groups are shown in Fig. 4J. Overall spine density was significantly higher in Nb62-r-mAb treated APP^NL-G-F^ mice compared to controls (*P* < 0.0001; Fig. 4K). Morphological analysis of spine subtypes showed that both thin and mushroom spines were significantly higher in Nb62-r-mAb mice (thin *P* < 0.0001; mushroom *P* < 0.01; Fig. 4L). To confirm the impact of Nb62-r-mAb on synapses, synaptic puncta were quantified after immunofluorescence labelling with presynaptic (Bassoon) and postsynaptic (PSD95) markers (Fig. 4M); pre- and postsynaptic markers were higher in Nb62-r-mAb treated mice compared to controls, the latter reaching significance (*P* = 0.0231; Fig. 4N).

In behavioural tests conducted immediately prior to sacrifice, Nb62-r-mAb treated mice performed significantly better than controls on all three tests, burrowing (*P* = 0.0041; Fig. 4O), Open Field (*P* = 0.0022; Fig. 4P) and NOR (*P* < 0.0001; Fig. 4Q) testing. These three tests interrogate different aspects of cognition: Burrowing exploits a natural rodent behaviour to test hippocampal integrity; Open Field tests exploratory behaviour, locomotor activity and anxiety, and has been reported as an early index of cognitive impairment in APP^NL-G-F^ mice^42^; NOR provides a simple test of learning and working memory, widely used in drug testing in AD models. Together, these results demonstrate that prolonged treatment with Nb62-r-mAb had a marked effect on cognition, reducing cognitive decline and rescuing other behavioural deficits in APP^NL-G-F^ mice.

## Discussion

Complement is an attractive therapeutic target in neurodegenerative diseases. Here, we provide proof-of-concept that an antibody blocker of the pro-inflammatory complement effector MAC can be delivered to the brain using an anti-TfR nanobody shuttle in a mouse model of AD. We demonstrate target engagement in brain, effective protection against neurodegeneration downstream of amyloid plaque formation and protection against cognitive decline in the model.

Drugs targeting complement are increasingly used for the treatment of systemic inflammatory diseases, but use in dementia requires efficient brain penetrance, lacking in current anti-complement drugs, most of which are mAbs with minimal BBB penetrance.^22^ To address this deficit, we generated a brain-penetrant anti-complement drug based on our novel anti-C7 mAb that specifically inhibits the assembly of MAC, the cytolytic complex of complement responsible for triggering inflammatory pathways, including the NLRP3 inflammasome, in diverse cell types.^43,44^ We targeted MAC because we have demonstrated that MAC drives neuroinflammation, neurodegeneration and synapse elimination in AD models.^38^ C7 is essential for MAC formation, less abundant than C5, the target of Eculizumab and other drugs in the clinic (∼2-fold lower plasma concentration) and is not an acute phase reactant,^45^ properties that make it a better drug target. Inhibiting C7 may also confer less infection risk compared to C5 because C5a-mediated neutrophil recruitment is unimpaired.^46^

The selected anti-C7 mAb, 73D1, an efficient cross-species inhibitor of C7 and of comparable efficacy to the benchmark C5-blocking mAb BB5.1 in models of peripheral inflammation,^27,47,48^ was used as template to generate a r-mAb tagged with a nanobody (Nb62) that binds TfR on brain endothelial cells to engage receptor-mediated transcytosis. Nb62 was shown to deliver a barrier-impenetrant neuropeptide into brain,^28^ and a modified version mediated brain delivery of anti-BACE1 mAb in mice expressing human TfR.^29^ Others have demonstrated the capacity of TfR shuttles to deliver antibodies and other large cargo into the brain.^49, 50^ We confirmed that Nb62-r-mAb administered systemically in healthy C7 deficient mice crossed the BBB; however, brain clearance of the agent was rapid, as previously reported for other TfR-delivered cargoes.^28,51,52^ Plasma levels of Nb62-r-mAb fell sharply over the 24-h period; we showed that this was a consequence of enhanced uptake of TfR-targeted cargo in the periphery, as reported elsewhere.^53,54^ The Nb62 nanobody was engineered for reduced TfR affinity to minimize peripheral uptake^28^; nevertheless, rapid clearance remained, highlighting the need for further modifications for efficient delivery and sustained impact.

The APP^NL-G-F^ mouse contains an endogenous promoter-driven single knock-in of human APP incorporating three familial AD-associated mutations that together promote toxicity and plaque formation by increasing Aβ production and aggregation; it is a robust model for studying plaques and glial responses to plaques but lacks other components of AD pathology. We selected this model based on practicalities; synapse loss, plaque accumulation and cognitive impairment occur relatively early and in a consistent manner. Importantly, we recently showed that complement activation products are markedly increased in APP^NL-G-F^ mice and increase further with age, evidence of increasing complement dysregulation with progressing pathology.^38^ Systemic C7 was first saturated with the parent mAb to ensure that the Nb62-r-mAb retained C7-binding capacity; we chose to use the parent mAb for this purpose rather than Nb62-r-mAb to reduce the risk of swamping the brain uptake pathway. Systemic delivery of Nb62-r-mAb over 7 days reduced brain levels of TCC, a marker for MAC formation, confirming target engagement; C3 fragments, markers of complement activation, were also significantly reduced, likely because MAC inhibition reduces injury and resultant complement activation, as previously shown in a demyelination model.^55^ Nb62-r-mAb also reduced Aβ_42_ levels in APP^NL-G-F^ brain, while hippocampal synaptic spine density was significantly increased. These results demonstrate a clear and rapid effect of shuttle delivery of anti-C7 r-mAb on neurodegeneration.

Having confirmed target engagement, we extended the treatment period to 13 weeks; Nb62-r-mAb treatment inhibited MAC formation, reduced brain inflammation and protected from complement-driven synapse loss, reflected in a significant increase in spine density that translated to improved cognition; the relationship between spine density and cognition has been reported in other mouse AD models.^56,57^ Notably, these effects of Nb62-r-mAb treatment occurred in the absence of an impact on the number of amyloid plaques, although Aβ_42_ levels in brain extracts were significantly reduced, implying an effect on plaque amyloid fibril content.^58^ While the role of complement and inflammation on amyloid fibril formation and plaque accumulation deserves further attention, our findings suggest that the beneficial effects on cognition are likely downstream of amyloid plaques; hence, plaque removal is not a prerequisite for synapse rescue and improved cognition. Indeed, others have reported that C3 deficiency improved cognition but increased plaque load in an AD model.^59^ Levels of inflammatory cytokines, including IL-1β, a marker of inflammasome activation, were markedly reduced in Nb62-r-mAb treated mice. Microglia express the NLRP3 inflammasome and are the primary source of IL-1β in brain,^60,61^ while we and others have shown that MAC directly triggers inflammasome activation in diverse cell types.^43,44^ Together, these observations suggest that activation of microglia by MAC contributes to neuroinflammation.

This work provides proof-of-concept that anti-complement drugs can be delivered to the brain using shuttles to reduce complement dysregulation, neuroinflammation and neurodegeneration, and confirm our recent demonstration that MAC is an important player in complement-mediated synapse loss in AD models.^38^ The work supports and extends our recent demonstration that treatment with the unmodified mAb 73D1 in 9-month-old APP^NL-G-F^ mice reduced brain complement activation, amyloid load and synapse loss.^62^ In this context, brain penetrance of the mAb was dependent on BBB impairment, seen only late in the disease course; therefore, developing a brain-penetrant molecule is essential for early treatment and prevention of AD in models and humans. Taken together, these studies consolidate our hypothesis that complement dysregulation, and specifically MAC formation, contribute to neuroinflammation and neurodegeneration in AD and the corollary that brain-targeted MAC inhibition may provide a novel therapeutic approach in AD and other dementias. While targeting complement always carries a risk of infection, MAC inhibition carries a much reduced risk compared with early pathway inhibition because it does not impair pathogen tagging and opsonization.^22,23^ For anti-C5 therapies already in the clinic, the risk of *Neisseria* infection is managed using vaccination and prophylactic antibiotics^22^; C7 inhibition would require a similar approach to prophylaxis.

The work has several limitations. First, we used a single AD mouse model, albeit one of the best for the study of amyloid pathology; replication in other models, including those with tau pathology, is now needed. Second, peripheral inhibition of C7 was necessary to enable the Nb62-r-mAb to access the brain unimpeded by cargo; we are currently exploring several strategies to eliminate the need for this pre-treatment by modifying the r-mAb and by testing recombinant forms of other human C7-blocking antibodies from our toolbox for optimal delivery. Third, we used male mice in all experiments, a pragmatic choice to minimize the effects of known sex differences in complement activity in mice on study outcomes; testing in females should be prioritized in future confirmatory studies. Finally, we do not include a comprehensive analysis of the impact of C7 inhibition on glial activation state or neuronal survival in the model; this work is ongoing.

## Supplementary Material

awae278_Supplementary_Data

## Data Availability

The data that support the findings of this study are available from the corresponding author, upon reasonable request.
