# Supplementary figures and images for: Brain-penetrant complement inhibition mitigates neurodegeneration in an Alzheimer’s disease mouse model
Source: Brain. 2024 Aug 31;148(3):941–54. doi: 10.1093/brain/awae278 (PMC11884734; doi:10.1093/brain/awae278)

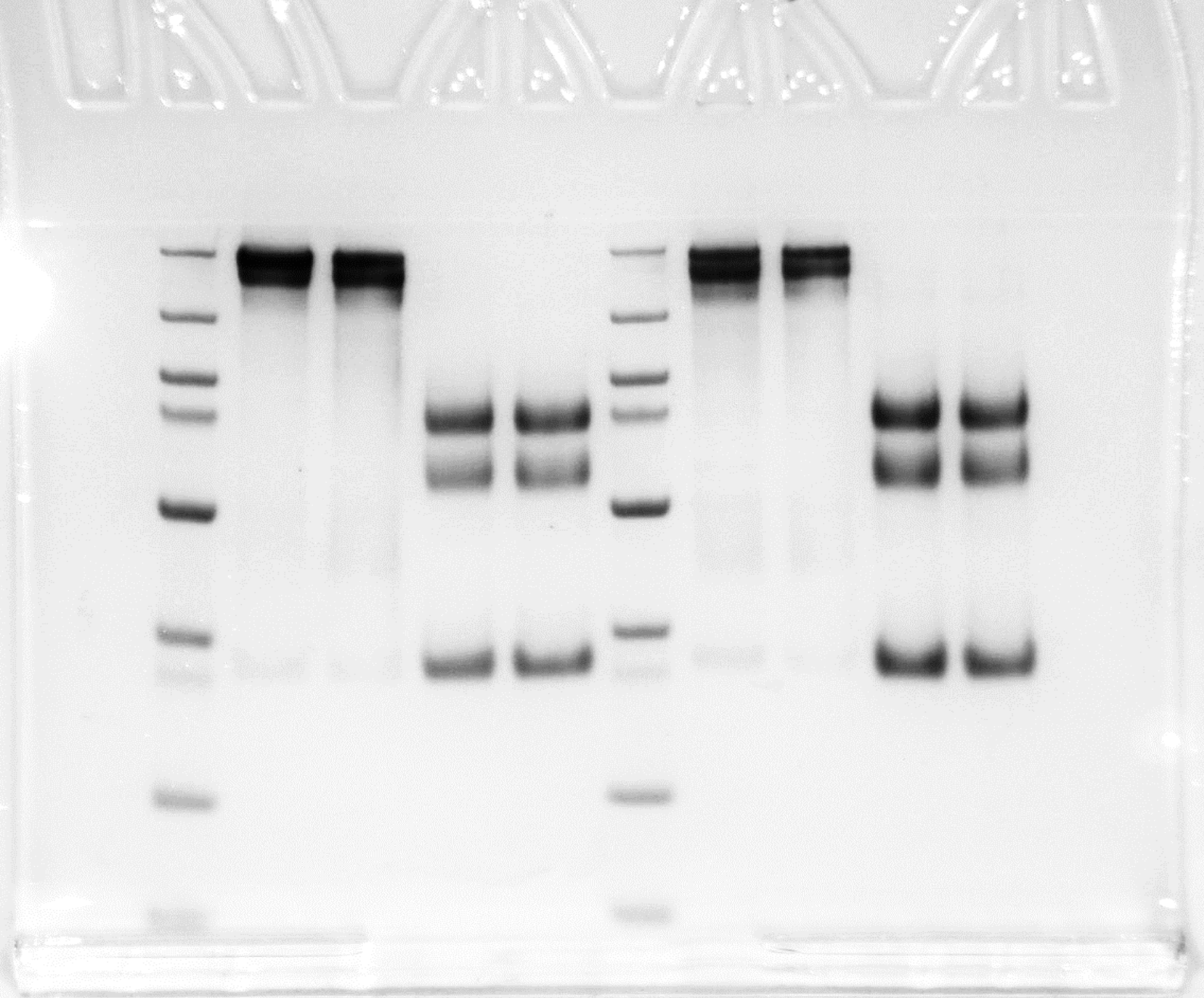

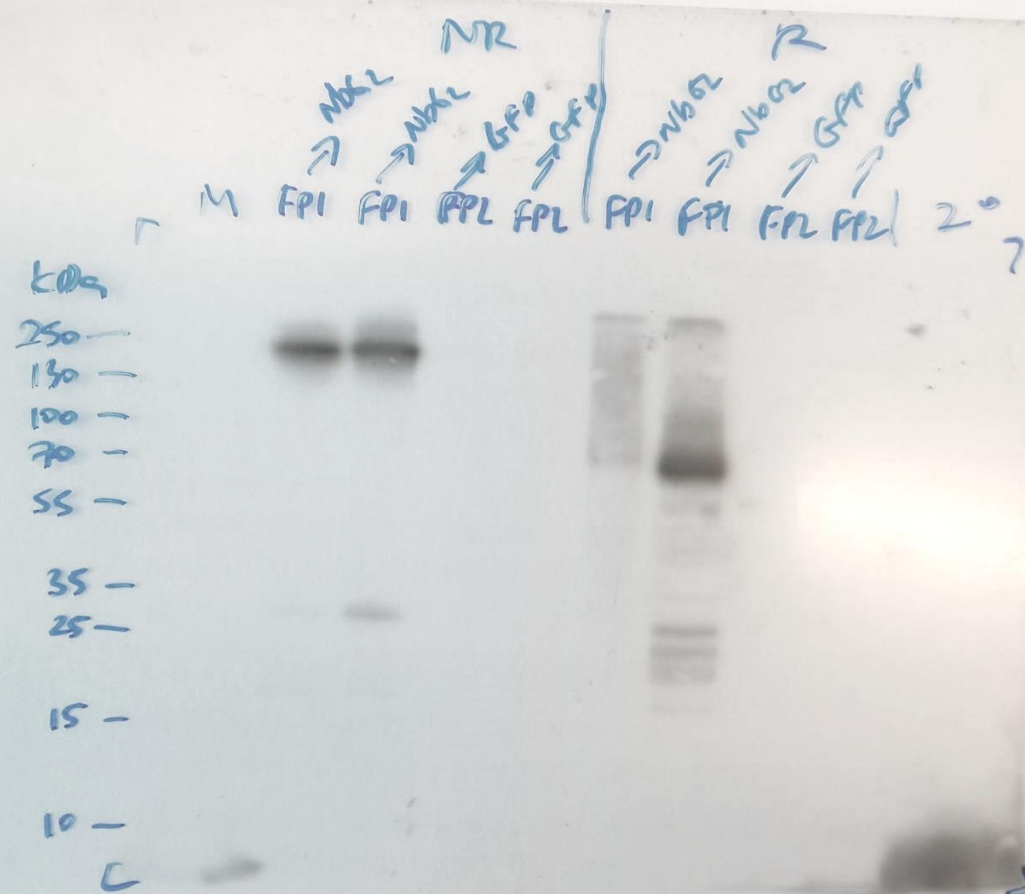

Supplement: awae278_Supplementary_Data [file awae278_supplementary_data.pdf]
